# Enhancing Underwater Images of a Bionic Horseshoe Crab Robot Using an Artificial Lateral Inhibition Network

**DOI:** 10.3390/s25051443

**Published:** 2025-02-27

**Authors:** Yuke Ma, Liang Zheng, Yan Piao, Yu Wang, Hui Yu

**Affiliations:** 1 School of Electronic and Information Engineering, Changchun University of Science and Technology, 7089 Weixing Road, Changchun 130022, China; frankma120816@163.com (Y.M.); piaoyan@cust.edu.cn (Y.P.); wangyulfy@cust.edu.cn (Y.W.); 2 School of the Electrical and Information Engineering, Jilin Agricultural Science and Technology University, 77 Hanlin Road, Jilin 132101, China; 3 School of Information and Control Engineering, Jilin Institute of Chemical Technology, Jilin 132022, China; yuhuielsa@foxmail.com

**Keywords:** bionic horseshoe crab robot (BHCR), multiple underwater movement, amphibious bionic robot, lateral inhibition

## Abstract

This paper proposes an underwater image enhancement technology based on an artificial lateral inhibition network (ALIN) generated in the compound eye of a bionic horseshoe crab robot (BHCR). The concept of a horizontal suppression network is applied to underwater image processing with the aim of achieving low energy consumption, high efficiency processing, and adaptability to limited computing resources. The lateral inhibition network has the effect of “enhancing the center and suppressing the surroundings”. In this paper, a pattern recognition algorithm is used to compare and analyze the images obtained by an artificial lateral inhibition network and eight main underwater enhancement algorithms (white balance, histogram equalization, multi-scale Retinex, and dark channel). Therefore, we can evaluate the application of the artificial lateral inhibition network in underwater image enhancement and the deficiency of the algorithm. The experimental results show that the ALIN plays an obvious role in enhancing the important information in underwater image processing technology. Compared with other algorithms, this algorithm can effectively improve the contrast between the highlight area and the shadow area in underwater image processing, solve the problem that the information of the characteristic points of the collected image is not prominent, and achieve the unique effect of suppressing the intensity of other pixel points without information. Finally, we conduct target recognition verification experiments to assess the ALIN’s performance in identifying targets underwater with the BHCR in static water environments. The experiments confirm that the BHCR can maneuver underwater using multiple degrees of freedom (MDOF) and successfully acquire underwater targets.

## 1. Introduction

With the increasing demand for autonomous underwater vehicles (AUVs) that possess high mobility, long endurance, energy efficiency, and stealth capabilities, researchers have been exploring various sources of inspiration [1,2]. The combination of bionic robots and optimization algorithms plays a crucial role in advancing coastal exploration [3,4,5,6,7,8,9]. Existing untethered bionic robots such as sea urchin soft robots [10], climbing soft robots [11], frog soft robots [12,13], fish robots [14,15,16,17], snake robots [18,19], salamander robots [1], dolphin robots [20] and turtle robots [21,22,23,24,25] have been developed by studying aquatic animals. These studies primarily focus on dynamic modeling [26], motion control [1,20,27,28], swimming efficiency [29] and perception [30], gradually propelling the advancement of underwater robot technology. However, capturing high-quality underwater images on a bionic robot platform is particularly challenging due to color distortion, brightness attenuation, snow noise caused by refraction from floating substances in water during light propagation, and the complexity involved in implementing bionic compound eyes. Therefore, it is highly significant to investigate underwater image enhancement using bionic compound eyes. This not only provides users with an improved visual experience but also has potential to enhance performance across various related underwater visual tasks.

Underwater images are plagued by quality defects such as color distortion, poor visibility, and blurred details. These phenomena can compound each other, further degrading image quality. Numerous studies have been conducted to enhance visual quality from various perspectives. Hartline’s comprehensive and systematic exploration of optic nerve electrical pulses in horseshoe crabs led to the discovery of many principles governing various visual system functions, earning him a Nobel Prize in 1967 [31]. Scientists have also made significant progress in visual enhancement research. Bianco et al. [32] found that water reduces light intensity across different colors while Chiang et al. [33] proposed combining classical sea fog removal algorithms with selective attenuation models for underwater light sources to effectively reduce their impact on image quality. Wen et al.’s method approximates underwater light scattering rates and background lighting by reversing clear and transparent underwater image models [34]. In 2016, Li et al.’s algorithm based on blue–green channel defogging and red channel correction was proposed as an effective means of recovering contrast loss and color distortion in underwater images [35]. With the development of image processing methods based on deep learning, a significant amount of research on underwater image enhancement methods based on deep learning has been conducted by domestic and foreign scholars. In 2024, an underwater image enhancement framework based on three stages was proposed by Hao et al., and virtual underwater image synthesis, underwater image depth map estimation, and reinforcement learning were used for underwater image enhancement to configure advanced physical models [36]. An underwater image perception fusion module was proposed by Shunmin An et al. from Tongji University, and experiments showed that this method could achieve better image enhancement results compared with existing underwater image enhancement methods [37]. A neural network named LiteEnhanceNet was introduced by Song Zhang et al. from China Agricultural University, and experiments showed that the algorithm achieved excellent running speed [38]. An unsupervised underwater image enhancement algorithm (HUFI-Net) specifically for this dataset was proposed by Zhen Li et al. from Yunnan University to correct the sharpness and color of images, and experiments have shown that this method is generalized and effective on UIEB and RUIE underwater image datasets [39]. An attention-based color-consistent underwater image enhancement network was proposed by Chang et al. from Shandong Technol & Business University. The method consists of three parts: lighting detail network, balanced stretching module, and predictive learning module, and experiments have shown that this method produces more natural enhanced images than other state-of-the-art methods [40]. However, these studies have only focused on the research of underwater image enhancement algorithms based on machine learning. The actual small AUV carries a limited power supply, and underwater image enhancement algorithms based on machine learning consume a lot of energy during operation. It is difficult or impossible to install underwater image enhancement algorithms based on machine learning on small AUV devices. Therefore, for small AUV devices, there are still many mysteries surrounding the vision system that need further investigation.

As an amphibious bionic robot, bionic horseshoe crab robots (BHCRs) equipped with water jet thrust exhibit superior swimming capabilities in comparison to traditional propeller underwater propulsion devices, showcasing high stability, effective sealing, exceptional visual characteristics, and reduced noise levels [41,42]. In the challenging marine environment, BHCRs integrated with sensors and instruments naturally emerge as a primary means for expanding human perception [43,44,45,46,47,48].

In this study, we developed a bionic eye by employing four contemporary infrared cameras with distinct perspectives to emulate the visual system of horseshoe crabs [49]. By utilizing four image enhancement algorithms, we compared and analyzed the feasibility of implementing BHCR lateral inhibition network in underwater imagery. Additionally, we designed a bionic compound eye using four perspective-diverse cameras to mimic the vision of squid [49]. Furthermore, we incorporated four ultraviolet sensors to replicate the biological ultraviolet sensing capability found in squids, enabling our bionic robot to perceive surrounding ultraviolet intensity through the compound eye system and achieve lateral inhibition vision in underwater environments.

The main contributions of this work are summarized as follows: (1) A new Limulus eye structure simulated by four HD cameras is proposed, which can effectively realize underwater target detection by combining it with a side-suppression image processing algorithm. (2) Based on the lateral suppression phenomenon of BHCR compound eye, an underwater image enhancement technique is proposed, aiming to introduce the concept of lateral suppression into underwater image processing. (3) In the application research of bionic Limulus amphibious robot, the experiments mainly focus on image processing and target recognition based on artificial side-suppression network. Experimental results show that the proposed algorithm can enhance the important information in underwater images. Compared with other algorithms, this algorithm can effectively deal with the problems of low contrast, high noise and large shadow in underwater images, achieving the effect of suppressing pixels in other positions.

The remainder of this paper is organized as followed. The artificial lateral inhibition network (ALIN) algorithm flow is discussed in Section 2. In Section 3, the algorithm constructed in this paper is compared and analyzed with several classical underwater image enhancement methods, and the corresponding visual tracking experiments are carried out. Section 4 discusses our experimental results and gives a conclusion.

## 2. Principle Description of an ALIN Algorithm

**Figure 1:**
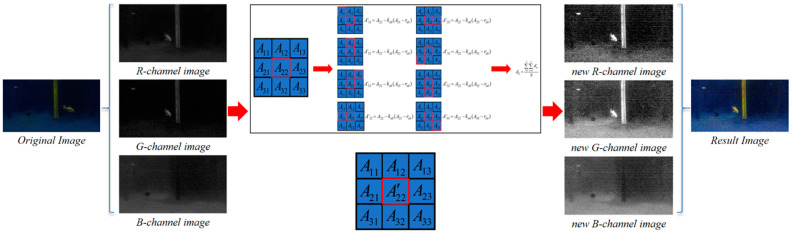
Schematic diagram of algorithm processing flow.

**Figure 2:**
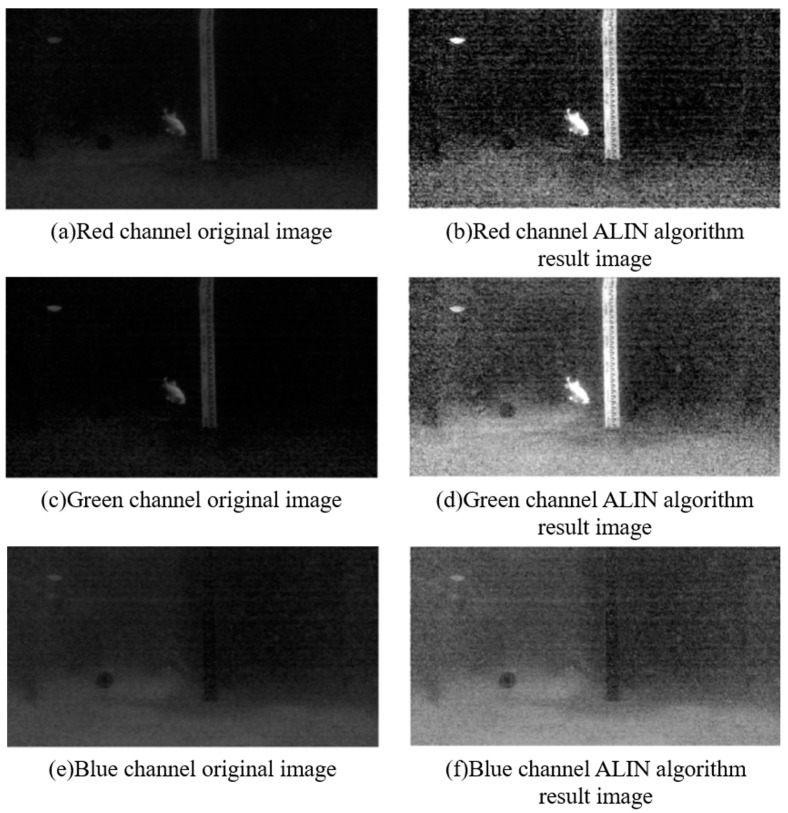
Comparison of the renderings of RGB channels before and after ALIN algorithm processing.

**Figure 3:**
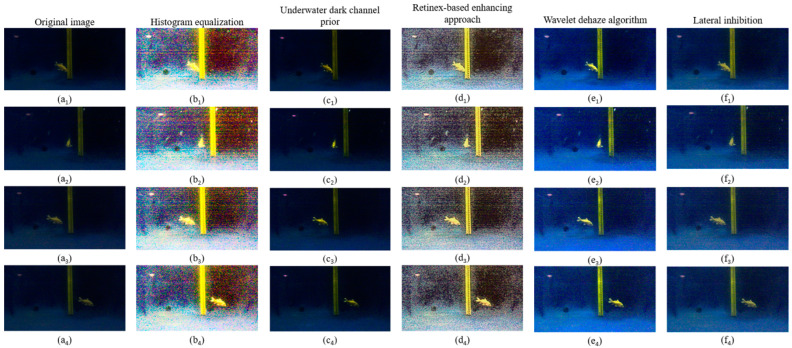
Comparison and analysis of different algorithms of underwater image acquisition. The original images of different positions of the target object in the process of moving are shown in (**a1**–**a4**); the histogram equalization result images of different positions of the target object in the process of moving are shown in (**b1**–**b4**); the dark channel prior algorithm results of different positions of the target object in the process of moving are shown in (**c1**–**c4**). The processing result images based on Retinex algorithm at different positions of the target object in the moving process are shown in (**d1**–**d4**); the processing result images based on wavelet de-fogging algorithm at different positions of the target object in the moving process are shown in (**e1**–**e4**); the processing result images based on ALIN algorithm at different positions of the target object in the moving process are shown in (**f1**–**f4**).

In this section, we present an ALIN algorithm that integrates theoretical analysis and mathematical modeling of the algorithm. First, we explain the theoretical basis of horizontal inhibition effect, which provides theoretical support for the rationality of the algorithm. Furthermore, we established the mathematical model of the ALIN algorithm and derived the lateral inhibition model based on the Retinex model, which introduces the lateral inhibition theory into the field of underwater image enhancement. The schematic diagram of the algorithm implementation process is shown in Figure 1. To illustrate the enhancement process of each channel in our artificial lateral inhibition network (ALIN) algorithm, we output separate enhancement images for each channel, as depicted in Figure 2. And then, we conducted a comparative analysis with four state-of-the-art underwater image enhancement (UIE) algorithms, including histogram equalization algorithm, underwater dark channel prior algorithm and wavelet dehaze algorithm, as shown in Figure 3.

In the algorithm implementation process, we first divide the image into three independent channels R, G and b, and then calculate the surrounding eight adjacent pixels by traversing each pixel value in each channel, and assign the calculation result to the new pixel value of the corresponding position of the channel. This step ensures that each pixel value is adjusted by a formula that reflects the side-suppression operation between pixels. After completing the above steps, we reassemble all the new pixel values into grayscale images for the R, G, and B channels. This process not only preserves the basic features of the image, but also further enhances the sharpness and contrast of the image. Finally, we combine the three newly exported channels to generate a new color image and display it through the output device. Overall, the ALIN algorithm we propose excels in theoretical analysis and mathematical model construction, capable of achieving fine processing and optimization of images, thereby contributing new strength to the development of the field of image processing.

When the visual nerve cells of the compound eye of the horseshoe crab are irradiated by strong light, the light enhances the photosensitivity induction effect on the visual cells of the nearby compound eye and inhibits the photosensitivity of the optic nerve cells that are relatively far away from the visual cells, achieving the effect of “enhancing the center and suppressing the surroundings”. The concept of a lateral inhibition network is derived from this theory. However, at present, there is no image processing algorithm that can completely replace the visual theory of lateral inhibition. How to use existing image processing algorithms to simulate the lateral inhibition algorithm is the first and most important problem to be solved.

Channel space LRN was first proposed and widely used in ALIEX NET. The pixel neighborhood defined by LRN is normalized on each individual image channel, and the depth of each pixel position is normalized. The formula is as follows:

(1) $$b_{x,y}^{i}=\frac{\alpha_{x,y}^{i}}{{\left[k+\alpha \sum_{j=\max\left(0,i-\frac{n}{2}\right)}^{\min\left(N-1,i+\frac{n}{2}\right)}{\left(\alpha_{x,y}^{j}\right)}^{2}\right]}^{\beta }}$$

(2) $$b_{x,y}^{k}=\frac{a_{x,y}^{k}}{{\left[k+\alpha \sum_{i=\max\left(0,x-\frac{n}{2}\right)}^{\min\left(W,x+\frac{n}{2}\right)}\sum_{i=\max\left(0,x-\frac{n}{2}\right)}^{\min\left(W,x+\frac{n}{2}\right)}{\left(a_{i,j}^{k}\right)}^{2}\right]}^{\beta }}$$

where i represents the output of filter, $\alpha_{x,y}$ and $b_{x,y}$ represent the pixel value of normalized (x,y) coordinate. N represents the total number of channels. k,α,β and n represent the hyper-parameter. 

(x,y) represents the corresponding pixel coordinate value in the image, $L_{d}\left(x,y\right)$ represents the intensity of light that is attenuated by its propagation through water, $L_{g}\left(x,y\right)$ represents the reflected light of the target in underwater optical imaging. When the light emitted by the light source propagates in water, part of the light enters the camera through the refraction of suspended particles, part of the light shines on the target and is directly reflected to the camera, and the other part of the light shines on the target and is reflected to the suspended particles and then refracted to the camera.

$\sqrt{{\left(m-p\right)}^{2}+{\left(n-q\right)}^{2}}$ is the distance between the camera and the ultraviolet sensor. The coefficient A represents the gain factor for a specific local region. $\sqrt{\frac{S_{1}^{2}+S_{2}^{2}}{2}}$ is the effective value of ultraviolet sensors. $F^{\prime }(m,n)$ is the value of the current pixel and F(m+i,n+j) is the value of the pixel near the current pixel. G(m,n) represents the final processing image.

To accurately simulate the lateral suppression algorithm and its performance using the current processing algorithm, we derive an ALIN mathematical model based on Retinex image model. In our ALIN algorithm, the inhibition coefficient is calculated

(3) $$G(m,n)=F^{’}(m,n)-\sum_{i=-l}^{l}\sum_{j=-l}^{l}k(S_{1},S_{2})F(m+i,n+j)$$

where $k\left(S_{1},S_{2}\right)$ represents the inhibition coefficient that base on ultraviolet value of current environment, $S_{1}$ and $S_{2}$ is the value of current environment which sensor from ultraviolet sensors. To fit the light value of the nonlinear underwater environment more truly, we use the singular value decomposition algorithm and the total least square method to fit the signal data collected by the ultraviolet sensor, to fit the environmental light condition. $k(S_{1},S_{2})$ is obtained by

(4) $$k(S_{1},S_{2})=A\sqrt{{\left(m-p\right)}^{2}+{\left(n-q\right)}^{2}}\sqrt{\frac{{S_{1}}^{2}+{S_{2}}^{2}}{2}}$$

where *A* is the hyperparameters, $\sqrt{{\left(m-p\right)}^{2}+{\left(n-q\right)}^{2}}$ is the distance between the camera and the ultraviolet sensor. $\sqrt{\frac{{S_{1}}^{2}+{S_{2}}^{2}}{2}}$ is the effective value of ultraviolet sensors.

To show the theoretical modeling of ALIN algorithm more clearly, the derivation process of the side inhibition coefficient and its physical meaning have been explained in detail.

STEP1: Define a local domain

Let the pixel intensity of the image at position (x,y) be denoted as I(x,y). A local region N(x,y), which contains an 3×3 pixel region centered on (x,y) is defined.

STEP2: Calculate the local mean intensity

(5) $$\bar{I}(x,y)=\frac{1}{9}\sum_{\left(u,v)\subset N(x,y\right)}I(u,v)$$

where the average pixel intensity in a local field is represented by $\bar{I}(x,y)$.

STEP3: Define side inhibition factor

The degree of suppression of local tie pixel intensity is controlled by the side-suppression coefficient α. The side inhibition coefficient is defined as.

(6) $$\alpha (x,y)=\frac{1}{1+\beta \cdot \bar{I}(x,y)}$$

where β, a positive constant used as a gain parameter, is utilized to adjust the intensity of the suppression. When the local mean intensity $\bar{I}(x,y)$ is increased, the side rejection factor α(x,y) decreases, thereby enhancing the perception of the main light signal.

STEP4: Application side inhibition factor

The pixel intensity I’(x,y) is adjusted by the side inhibition factor.

(7) $$I’(x,y)=I(x,y)-\alpha (x,y)\cdot \bar{I}(x,y)$$

The pixel intensity I(x,y) will be more prominently highlighted after side-suppression processing, while background noise and detail blur will be suppressed.

The physical significance of the lateral rejection coefficient α(x,y) is that the perception of the main signal is enhanced by adjusting the degree of suppression of the local average intensity. Specifically, it operates in two ways: First, the main signal is strengthened. When the local mean intensity $\bar{I}(x,y)$ is high, the side inhibition factor α(x,y) decreases, thus reducing the suppression of the main signal and making it more prominent. This helps to enhance detail in high-contrast areas, resulting in a sharper main body of the image. Second, background noise is suppressed. When the local mean intensity $\bar{I}(x,y)$ is low, the side-suppression coefficient α(x,y) increases, thus enhancing noise suppression and reducing the detail blur of the image. This helps to reduce noisy pixels in low-contrast areas, resulting in a smoother background part of the image. Through this mechanism, the main pixel information is better retained by the ALIN algorithm when processing images, while noisy pixels and image detail blur are suppressed, thereby improving image contrast and detail clarity.

To obtain the ambient light information accurately, we added a Kalman filter to the model to process the light information obtained by the ultraviolet sensor. Therefore, we established the state equation and observation equation related to BHCR.

To more accurately model the nonlinear underwater environmental illumination values, we utilized the singular value decomposition algorithm and global least squares method to fit the signal data collected by the ultraviolet sensor, thereby modeling the ambient illumination conditions.

## 3. Experiments

In this section, we conduct a series of experiments to prove the performance of ALIN algorithm. The ALIN algorithm proposed by us is applied to underwater images in UIE dataset. We compare the horizontal suppression optimization algorithm with histogram equalization, the multi-scale Retinex algorithm, and the dark channel algorithm, and present the performance evaluation results of the horizontal suppression optimization algorithm. Finally, we use ALIN algorithm to carry out target tracking experiment.

**Figure 4:**
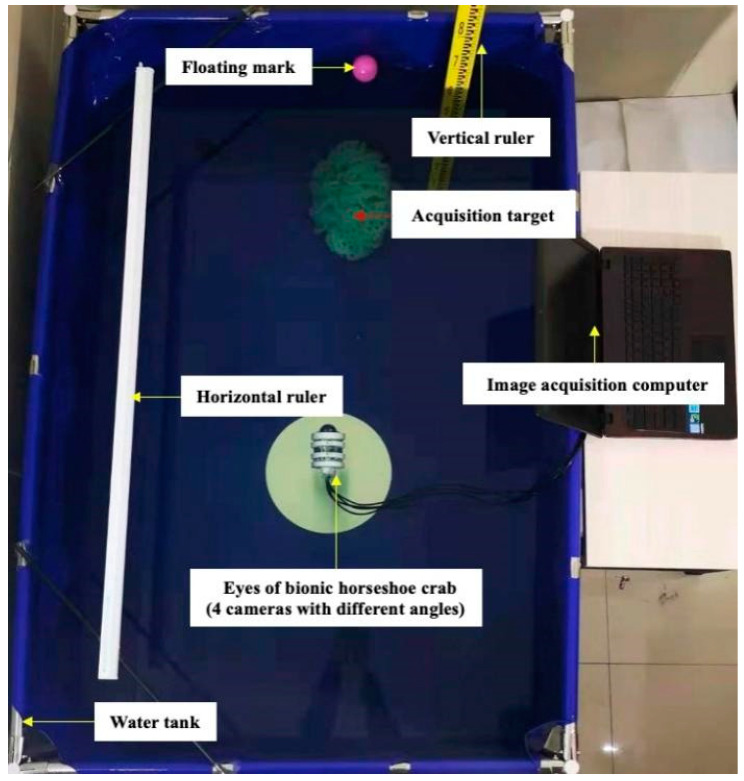
Experimental setup of underwater image acquisition.

We tested our ALIN algorithm on the RUIE dataset and the pool image collection dataset. The RUIE dataset contains 890 real-world underwater images. These images have been degraded to varying degrees. Due to the serious attenuation of the red channel in the underwater environment, these images show a blue–green tone [50]. To further verify the effectiveness of the ALIN algorithm proposed by us, we also used the pool acquisition image dataset for testing. The dataset was collected in a water tank 5 m long, 2 m wide, 1 m high and 0.9 m deep, as shown in Figure 4. The images in the dataset include near-range target images and long-distance target images captured by multiple cameras at the same time.

We compare ALIN algorithm with 8 state-of-the-art UIE algorithms, including histogram equalization algorithm, multi-scale Retinex algorithm and dark channel prior algorithm. We use code that is already publicly available to build these classical algorithms. The mathematical models of these three classical algorithms are shown in Table 1.

**Figure 5:**
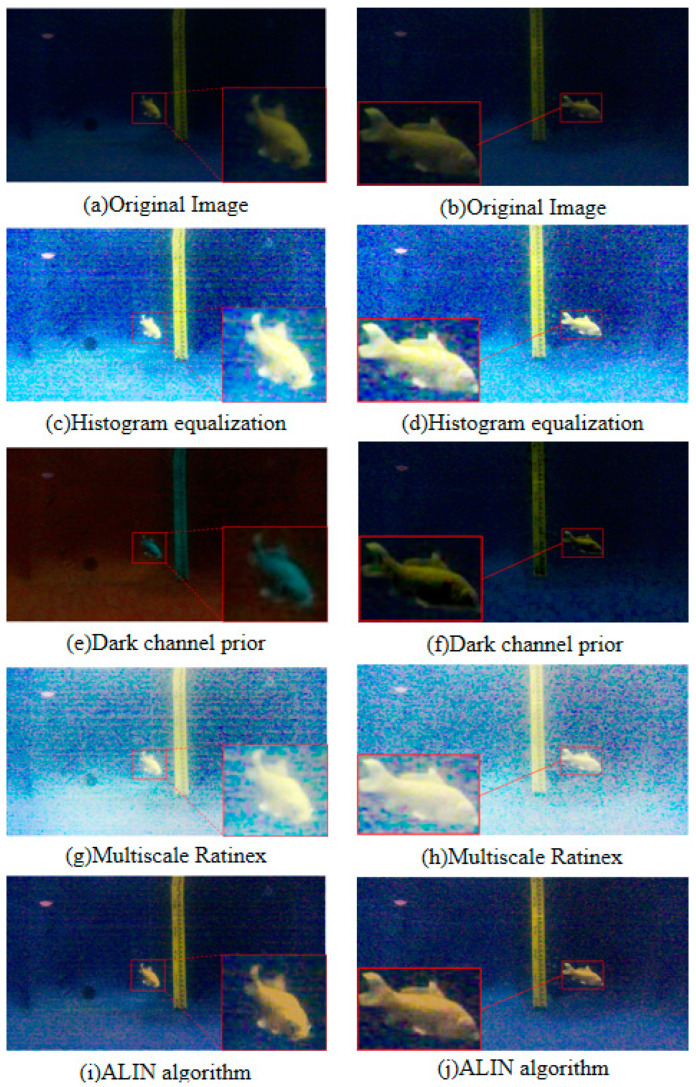
Five image enhancement algorithms are used to process underwater images acquired at close range. From left to right, there are region diagrams, histogram equalization diagrams, dark channel prior diagrams, multi-scale MSR diagrams and ALIN algorithms.

Firstly, we demonstrate the enhancement results of four different algorithms on a real underwater image. Phenomena such as color deviation, decreased contrast, and loss of detail are evident in the original image. As shown in Figure 5, histogram equalization, dark channel prior algorithm, and ALIN algorithm can effectively enhance the red channel in the original image. However, the MSR algorithm fails to correct the color deviation, specifically, it does not enhance the loss in the red channel, and it even reduces detail and visibility.

In the test of the RUIE underwater image dataset, a PSNR of 20.9 dB and an SSIM of 0.72 were achieved by the histogram equalization algorithm, which is relatively weak. This is mainly because its global adjustment method is difficult to effectively recover details in a complex underwater environment. A PSNR of 21.3 dB and an SSIM of 0.74 were achieved by the dark channel prior algorithm. Although it has certain advantages in fog removal, it is limited by scattering characteristics in the underwater environment and has insufficient detail retention. A PSNR of 22.0 dB and an SSIM of 0.76 were achieved by the multi-scale Retinex algorithm, which can improve contrast and detail by decomposing illumination and reflection components, but its computational complexity is relatively high. The wavelet de-fogging algorithm achieved a PSNR of 21.6 dB and an SSIM of 0.73, using the multi-resolution feature of wavelet transform to restore details, but its structural integrity still needs to be improved. In contrast, a PSNR of 22.9 dB and an SSIM of 0.79 were achieved by the ALIN algorithm. With the features of the outburst center and edge suppression of the side-suppression principle, the ALIN algorithm performs well in complex underwater environments, especially in the aspects of detail and structure preservation.

To better demonstrate the performance of our ALIN method in terms of algorithmic complexity, we selected some images from public underwater image datasets for image enhancement processing. Table 2 presents the evaluation of images from the RUIE dataset using subjective evaluation metrics.

**Figure 6:**
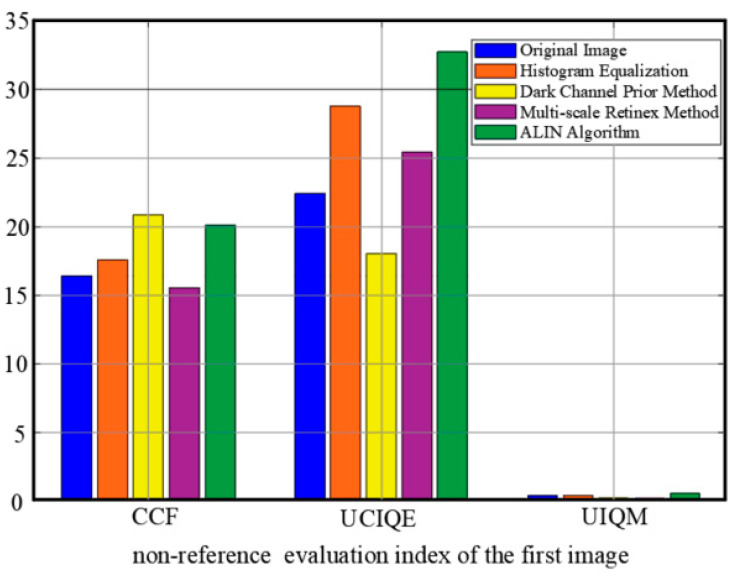
The full reference image quality evaluation index value of each algorithm, CCF, UCIQE and UIQM.

**Figure 7:**
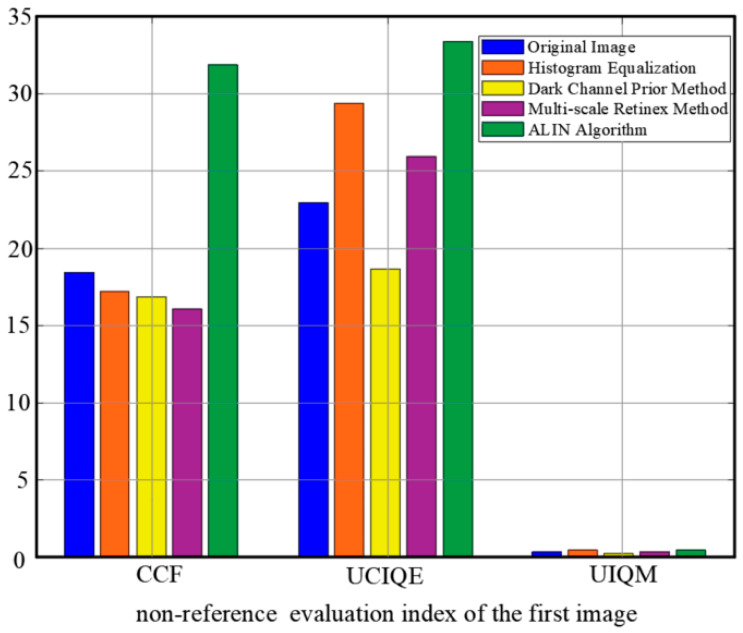
The other image fully references the image quality evaluation index values of each algorithm, CCF, UCIQE and UIQM.

To comprehensively evaluate the performance of underwater image enhancement algorithms, three commonly used Underwater Image Quality Evaluation indicators are employed: Color Constancy Fidelity (CCF), Underwater Image Quality Evaluation (UCIQE), and Underwater Image Quality Index (UIQM). These indicators measure image quality from different perspectives and can provide an objective evaluation of the algorithm’s performance, as shown in Figure 6 and Figure 7.

Color fidelity (CCF) is used to measure the performance of an image in terms of color reproduction. The accuracy of the color is judged by evaluating how close the color distribution of the image is to the real scene. The closer the CCF value is to 1, the higher the color fidelity of the image and the smaller the color distortion.

The Underwater Image Quality Evaluation Index (UCIQE) is a kind of nonreference quality evaluation index specially designed for underwater images. Many factors such as contrast, brightness, and color saturation of underwater images are taken into account, and the visual quality of images can be fully reflected.

The Underwater Image Quality Index (UIQM) is a comprehensive quality assessment indicator that measures image quality through three sub-modules: Colorfulness, Contrast, and Luminance. The scores of these three components are weighted and summed to give an overall quality score. The higher the UIQM value, the better the overall quality of the image.

**Figure 8:**
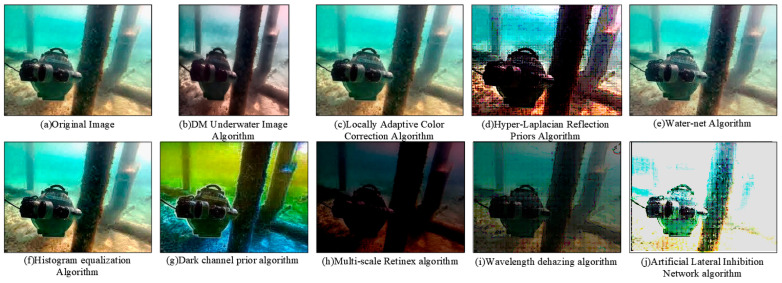
Image 1 presentation of the results of the RUIE underwater image dataset.

**Figure 9:**
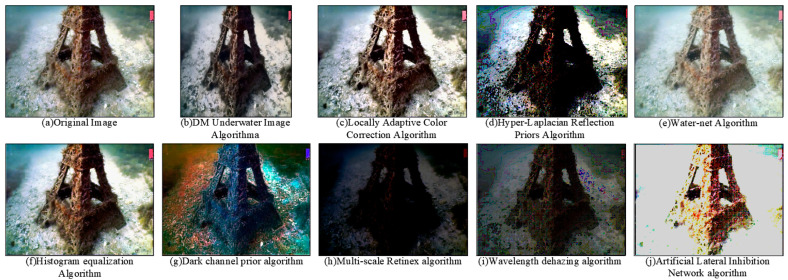
Image 2 presentation of the results of the RUIE underwater image dataset.

The key results of the experiment demonstrate that significant advantages in underwater image enhancement are shown by the ALIN method. Firstly, in terms of image quality evaluation, the highest UIQM index is reached by ALIN, indicating that in comprehensive performance such as color restoration, contrast enhancement, and detail retention, ALIN is superior to other methods, which have targeted optimization for image degradation caused by light attenuation and suspended particles in underwater scenes. Secondly, although in the UCIQE index, ALIN has similar performance to the dark channel prior method, while ensuring visual quality, computational efficiency is greatly improved by ALIN (as shown in Table 3, the shortest processing time is achieved by ALIN), which is crucial for underwater robots or dynamic tracking tasks with high real-time requirements. In addition, the universality of the method is further verified by the test results based on the RUIE dataset (Figure 8 and Figure 9): clearer subjective visual effects and more obvious separation of target details from the background are achieved by ALIN-processed images, especially in complex underwater lighting and cloudy environments, and its enhanced effect can effectively support subsequent target detection and tracking. It is worth noting that ALIN’s high efficiency (shortest processing time) stems from the lightweight design of its algorithmic structure, which gives it practical application potential in resource-constrained embedded platforms such as OpenMV, as shown in Table 4. By combining subjective and objective evaluation and real-time performance, the trade-off problem between speed and quality of traditional methods is solved by ALIN, and a reliable technical path for the engineering deployment of underwater real-time vision systems is provided. Its adaptability in dynamic target tracking and multi-sensor fusion scenarios can be further explored in the future.

During the operation of the robot, the camera module is responsible for capturing the raw image data of the robot. The experiment takes place in a water tank measuring 5 m in length, 2 m in width, and 1 m in height, with a water depth of 0.9 m. The experimental setup includes precise measuring horizontal and vertical rulers, as well as a laptop connected to a camera module that mimics pupil function for capturing images.

To comprehensively evaluate the performance of the algorithm, we choose two images with different features: one is an image of an underwater robot taken at close range, and the other is an image of the underwater Eiffel Tower. Figure 8a and Figure 9a show raw underwater images of these two scenarios.

Subsequently, we applied multiple algorithms to process the images. The histogram equalization algorithm effectively distinguishes well-lit areas from shadow areas when processing close-range images, but may cause color distortion and a yellowish tint in distant images. The dark channel prior algorithm performs well in removing water haze but may introduce a greenish shift in the images. The multi-scale Retinex algorithm (MSR) results in an overall greenish color cast, making it difficult to differentiate the water body. The ALIN algorithm shows significant effects in processing both short-range and long-range images but may introduce overall noise and color deviation from the original images.

**Figure 10:**
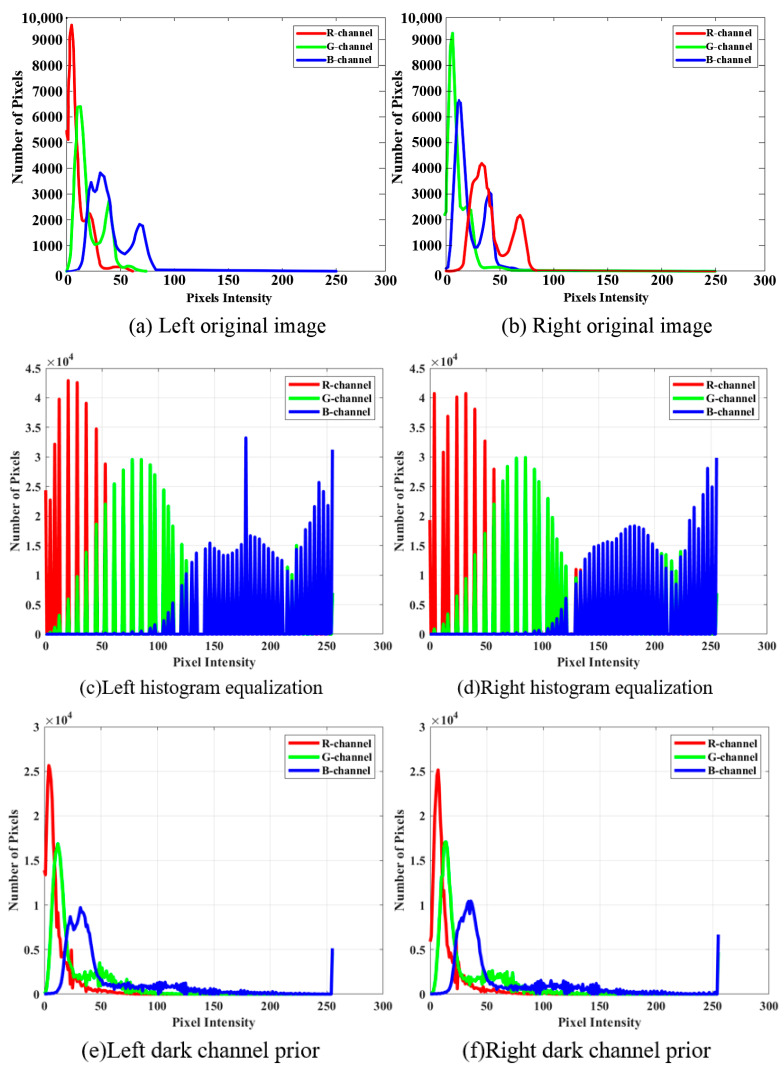

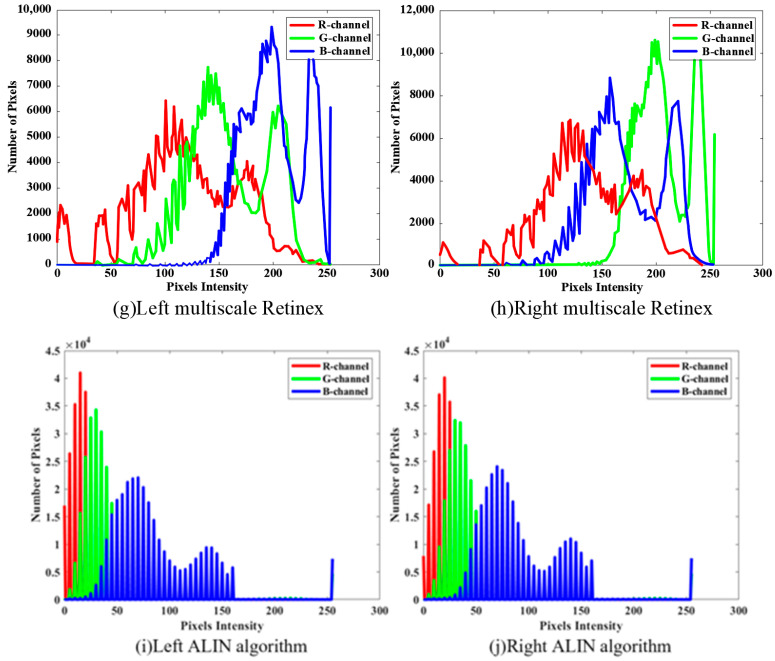
The data graph of R, G and B channels of the close-up image. From left to right are the original image, Histogram equalization algorithm, dark channel prior algorithm, multi-scale MSR algorithm and artificial lateral inhibition network algorithm (ALIN).

Further analysis of the R, G, B channel histograms of the processed underwater close-range images using various algorithms in Figure 10 reveals that the histogram equalization algorithm concentrates the blue and green channel data, with less noticeable improvement in the red channel. The dark channel algorithm concentrates the data in all three channels, with a higher volume of data in the green channel. In the MSR algorithm, the data in the three channels are concentrated in the middle, but the left part of the red channel is distorted. For the ALIN algorithm, most of the data in all three channels are concentrated on the right side of the image, leading to an overall red color bias and significant color distortion.

In summary, although the ALIN algorithm shows better color restoration in individual color channels compared to traditional algorithms, the overall color distortion due to the rightward color shift in all three channels. Therefore, further optimization of the algorithm is needed in future research to achieve more accurate image color restoration.

**Figure 11:**
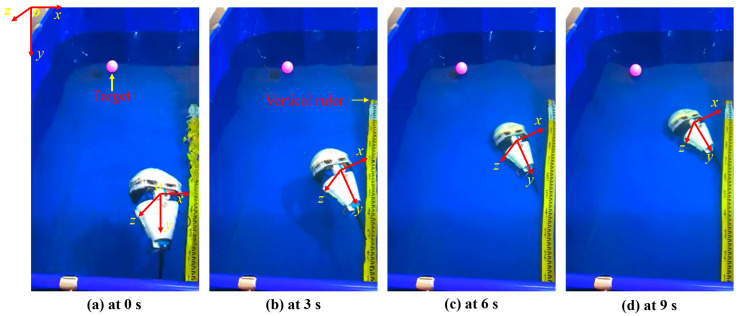
Tracking target movement scenario.

**Figure 12:**
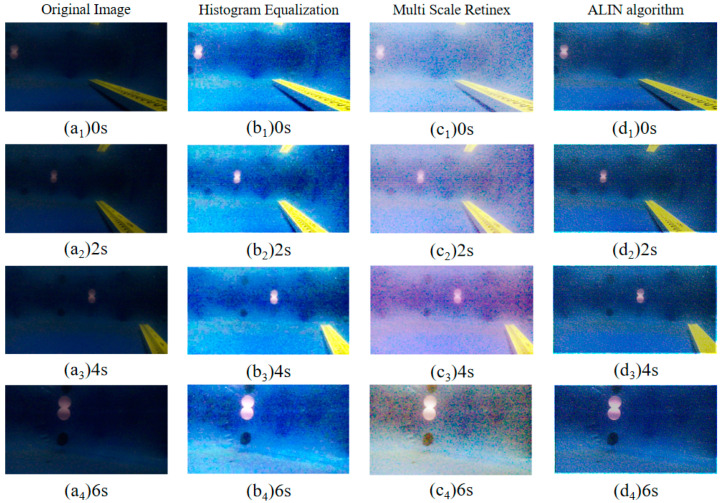
Result images at different time points during object tracking experiment with four different image enhancement algorithms.

**Figure 13:**
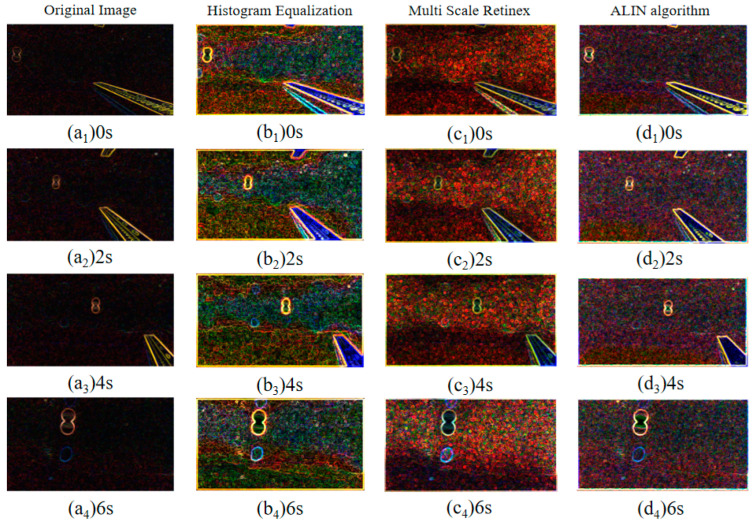
Resultant images of object recognition processing using Canny operator on various image enhancement algorithms.

In tracking motion experiments, we also use an identical experimental environment with artificial lateral inhibition network primarily to realize the tracking of fixed targets using a visual servo and to lay the experimental and theoretical foundation for the cooperation of multiple BHCRs. To simulate the observation signal and the image information collected by OpenMV, the collected dynamic image information can be used to achieve target acquisition. The objective of the experiment is to achieve static target tracking in the vertical direction (using the pink float as the target color for calibration). As shown in Figure 11, fuzzy algorithm is used to control the direction of the four BHCR waterjet propellers, and the direction of movement is adjusted synchronously to achieve the purpose of target tracking. Figure 11 shows the real-time images collected at 0, 3, 6 and 9 points during the robot’s movement, where the red rectangle is marked as the pink target to be identified. Figure 12 and Figure 13 are the underwater images from the robot’s perspective processed by the algorithm. Figure 12(d_1_–d_4_) is the image processed by ALIN algorithm. It can be seen that the image processed by ALIN algorithm enhances the ruler and target part, while suppressing the background part. Figure 13 shows the image processed by the pattern recognition algorithm. It can be clearly seen that the target and scale to be identified are shown as a white outline in the image, and the background area is shown as a black area.

The key results of the experiment are primarily demonstrated in the following three aspects: Firstly, the remarkable image enhancement effect is achieved based on the ALIN algorithm. As shown in Figure 12, the scale and pink floating object in the target area are effectively enhanced, and the background noise is significantly suppressed. This processing makes the target features more prominent in the complex underwater light environment, and provides visual input with a high signal-to-noise ratio for the follow-up tracking algorithm. Secondly, the effect of target extraction is further optimized by the pattern recognition algorithm. As shown in Figure 13, accurate segmentation of the target and environment is realized by binarizing the target contour into a white region and setting the background to black. This processing not only reduces the computational complexity, but also enhances the system’s robustness to the target shape, especially for the underwater edge blur scene caused by light scattering. Finally, the validation of the fuzzy algorithm for the four-propeller cooperative control is visually presented by the sequential images in Figure 11: Among the four moving nodes 0, 3, 6, and 9, the target marked by a red rectangle is always stably located in the center area of the image, indicating that static target tracking with sub-pixel accuracy in the vertical direction can be achieved by dynamically adjusting the direction of the water jet. These results not only verify the closed-loop effectiveness of the algorithm chain (ALIN pre-processing, pattern recognition, fuzzy control), but more importantly, through the deep coupling of target enhancement, feature extraction, and motion control, a scalable technical framework for collaborative positioning and task assignment of multiple BHCR systems is provided, especially in underwater collaborative work scenarios. This scheme, based on the combination of color characteristics and motion servo, can effectively address the common challenges caused by suspended particle interference and water flow disturbance.

## 4. Discussion and Conclusions

Based on the principle of image enhancement, this paper separately analyzes the existing problems of underwater image enhancement algorithms, as well as the causes and formation conditions of each algorithm. Using biomimetic Limulus technology, this paper introduces the main concepts of lateral inhibition theory, constructs the mathematical model of the lateral inhibition principle, and designs the ALIN algorithm based on this model. Next, this paper compares the ALIN algorithm with traditional image enhancement algorithms through experiments and simulations to verify its effectiveness in image enhancement. Experiments show that the ALIN algorithm can effectively enhance acquired images by highlighting image content in the central region and suppressing content in the surrounding regions. Next, by combining ALIN algorithm with traditional image enhancement algorithm, this paper compares and analyzes the influence of ALIN algorithm on traditional image enhancement algorithm. Experiments show that ALIN algorithm combined with Retinex algorithm has better effect and can better restore the content of reference image.

In this paper, a bionic horseshoe crab robot used artificial lateral inhibition network modelling and an image recognition algorithm to achieve underwater image processing and identification. The results verified that BHCR can use visual servos to achieve underwater dynamic target tracking. The main contribution of this paper is to use the characteristics of bionic and image processing algorithms to compare the advantages of eye target recognition technology in the BHCR. Combined with four image enhancement algorithms (white balance, histogram equalization, Retinex, and dark channel), the method can detect the edge of the lateral inhibition network for underwater objects with an obvious enhancement effect, which confirms that the lateral inhibition network can play a role in underwater image enhancement technology. Finally, the underwater target recognition experiment verifies the effectiveness of the lateral inhibition network algorithm for target recognition, and give the final vertical path and the deflection curve.

Future studies, including the underwater communication of multi-BHCRs and co-operation to complete the optimal path planning and target detection, will become the next research focus. In the multi-BHCR movement control algorithm, due to the special underwater environment, research on the high-precision underwater movement control algorithm should be the next urgent problem to be solved.

## Figures and Tables

**Table 1:** The mathematical formulations of four classical algorithms.

Algorithm	Mathematical Model	Algorithm Specification
Histogram Equalization	$P_{f}=\frac{1}{M\times N}\cdot h_{f},P_{a}=\sum_{k=0}^{k}P_{f},f(k)=255\cdot P_{a}\left(k\right)$	M×N is the number of pixels in the original image. $h_{f}$ is the number of pixels of each gray level after statistical mapping. $P_{f}$ is the normalized probability expression of the new gray statistical histogram. The variable $P_{a}$ denotes the ratio of pixels with values lower than k to the total number of pixels. f(k) is the final image.
Multi-Scale Retinex (MSR)	$r\left(x,y\right)=\sum_{k}^{K}w_{k}\left\{\log S\left(x,y\right)-\log\left[F_{k}\left(x,y\right)*S\left(x,y\right)\right]\right\}$	$F_{k}(x,y)$ is original input image, and S(x,y) is the filter function. $w_{k}$ is the weight of each scale. r(x,y) is the output image.
Dark Channel Prior (DCP)	$I\left(x\right)=J\left(x\right)t\left(x\right)+A\left(1-t\left(x\right)\right)$	J(x) indicated the image to be recovered. t is the transmittance, and A is the global illumination value. I(x) is the output image.
Artificial Lateral Inhibition Network (ALIN)	$G(m,n)=F’(m,n)-\sum_{i=-l}^{l}\sum_{j=-l}^{l}A\sqrt{{\left(m-p\right)}^{2}+{\left(n-p\right)}^{2}}\sqrt{\frac{S_{1}^{2}+S_{2}^{2}}{2}}F\left(m+i,n+j\right)$	$\sqrt{{\left(m-p\right)}^{2}+{\left(n-q\right)}^{2}}$ is the distance between the camera and the ultraviolet sensor. The coefficient A represents the gain factor for a specific local region. $\sqrt{\frac{S_{1}^{2}+S_{2}^{2}}{2}}$ is the effective value of ultraviolet sensors. $F^{\prime }(m,n)$ is the value of the current pixel and F(m+i,n+j) is the value of the pixel near the current pixel. G(m,n) represents the final processing image.

**Table 2:** SSIM and PSNR scores based on RUIE underwater image dataset.

Algorithm Name	PSNR (dB)	SSIM
Histogram equalization algorithm	20.9	0.72
Dark channel prior algorithm	21.3	0.74
Multi-scale Retinex algorithm	22.0	0.76
Wavelength dehazing algorithm	21.6	0.73
Artificial lateral inhibition network algorithm (ALIN)	22.9	0.79

**Table 3:** RUIE underwater image dataset results Image subjective evaluation data.

Algorithm Name	CCF	UCIQE	UIQM
Histogram equalization algorithm	22.6381	22.8653	0.2479
Dark channel prior algorithm	49.2690	36.4897	0.8117
Multi-scale Retinex algorithm	43.5015	Nan	−0.9137
Wavelength dehazing algorithm	64.0478	Nan	0.9416
Artificial lateral inhibition network algorithm (ALIN)	37.5225	36.3283	1.2224

**Table 4:** Comparative experimental data on algorithm runtime.

Algorithm Name	Running Time
Histogram equalization algorithm	3.009 s
Dark channel prior algorithm	2.6283 s
Multi-scale Retinex algorithm	2.5195 s
Wavelength dehazing algorithm	3.876 s
Artificial lateral inhibition network algorithm (ALIN)	1.785 s

## Data Availability

Due to the nature of this research, participants of this study did not agree for their data to be shared publicly, so supporting data is not available.
